# Do surveys with paper and electronic devices differ in quality and cost? Experience from the Rufiji Health and demographic surveillance system in Tanzania

**DOI:** 10.1080/16549716.2017.1387984

**Published:** 2017-11-20

**Authors:** Oscar Mukasa, Hildegalda P. Mushi, Nicolas Maire, Amanda Ross, Don de Savigny

**Affiliations:** ^a^ Impact evaluation Thematic Section, Ifakara Health Institute (IHI), Dar es Salaam, Tanzania; ^b^ Department of Epidemiology and Public Health, Swiss Tropical and Public Health Institute (Swiss TPH), University of Basel, Basel, Switzerland

**Keywords:** data management, health information systems, public health, health and demographic surveillance systems

## Abstract

**Background**: Data entry at the point of collection using mobile electronic devices may make data-handling processes more efficient and cost-effective, but there is little literature to document and quantify gains, especially for longitudinal surveillance systems.

**Objective**: To examine the potential of mobile electronic devices compared with paper-based tools in health data collection.

**Methods**: Using data from 961 households from the Rufiji Household and Demographic Survey in Tanzania, the quality and costs of data collected on paper forms and electronic devices were compared. We also documented, using qualitative approaches, field workers, whom we called ‘enumerators’, and households’ members on the use of both methods. Existing administrative records were combined with logistics expenditure measured directly from comparison households to approximate annual costs per 1,000 households surveyed.

**Results**: Errors were detected in 17% (166) of households for the paper records and 2% (15) for the electronic records (p < 0.001). There were differences in the types of errors (p = 0.03). Of the errors occurring, a higher proportion were due to accuracy in paper surveys (79%, 95% CI: 72%, 86%) compared with electronic surveys (58%, 95% CI: 29%, 87%). Errors in electronic surveys were more likely to be related to completeness (32%, 95% CI 12%, 56%) than in paper surveys (11%, 95% CI: 7%, 17%).The median duration of the interviews (‘enumeration’), per household was 9.4 minutes (90% central range 6.4, 12.2) for paper and 8.3 (6.1, 12.0) for electronic surveys (p = 0.001). Surveys using electronic tools, compared with paper-based tools, were less costly by 28% for recurrent and 19% for total costs. Although there were technical problems with electronic devices, there was good acceptance of both methods by enumerators and members of the community.

**Conclusions**: Our findings support the use of mobile electronic devices for large-scale longitudinal surveys in resource-limited settings.

## Background

The quality and cost of data are of concern in health information systems (HIS), particularly in resource-limited settings [1–3]. Reliance on data that are incomplete, inaccurate, or outdated may jeopardize decisions and risk the health of the population served by the health system (HS). Health and Demographic Surveillance Systems (HDSS) are making available health-related household based longitudinal data to support the HS in low- and middle-income countries (LMICs) [4–14]. However, there is a need to ensure that the quality of data is high and that costs are minimized. Investing in efforts to evaluate interventions with the potential to improve the efficiency of HDSS surveys is important. Mobile electronic survey tools are among the options for consideration because they have been applied and evaluated in health surveys other than HDSS. In 1991, Forster et al. [15] in the Gambia were among the first to apply digital survey methods. They reported a 31% shorter enumeration time using electronic compared with paper questionnaires, and later in Tanzania, in 2005, a personal digital assistant (PDA) model of electronic devices was shown to achieve over 99% data completeness in a cross-sectional survey of over 21,000 rural households in southern Tanzania [16]. Initial analyses were possible within 24 hours after the last day of the survey, and there were no noteworthy experiences of device-related problems or data loss. In Oceania, Yu et al. [17] reported completely error-free data from electronic compared with 21% records with errors in the paper-based dataset. In the same study, data entry at the point of capture eliminated 93% of an estimated 20.5 hours in data entry, validation, and cleaning processes compared with the paper-based system. Other studies have also contributed evidence on potential benefits of electronic over paper tools in health surveys including an improved time efficiency [18,19] and a greater ability to reconstruct the field interviewers’ daily activities for quality control [20]. All these findings, however, were from standalone cross-sectional survey experiences. Since there is a scarcity of evidence on the potential of electronic data collection for continuous longitudinal surveillance such as in HDSS settings, we report a comparison of data collection using paper and electronic tools in the Rufiji HDSS, one of the long-established longitudinal household based surveillance systems in Tanzania. We assessed the quality and cost of data collection, as well as the acceptance of the survey methods by enumerators and survey respondents.

## Methods

### Rufiji HDSS

Established in 1998, the Rufiji Demographic Surveillance System (RDSS) (Member of the International Network of Field Sites with Continuous Demographic Evaluation of Populations and their Health in Developing Countries (INDEPTH) http://www.indepth-network.org/, one out of 38 so far participating in the network, from 19 different countries) is one of the longitudinal household based data platforms supporting the Ministry of Health in Tanzania [21]. In 2008, it operated in 31 villages, covered an area of 1,813 km^2^, and had 16,427 active households. The Rufiji demographic surveillance area is in Rufiji District, 178 km south of the commercial capital city, Dar es Salaam. In accordance to general procedures, HDSS operations begin with an initial census of the population and proceed with monitoring vital events (births, deaths), migration, selected health outcomes, and other demographic and lifestyle variables. Control household visits are carried out to check the completeness of the follow-up procedures. The household is the basic survey unit and is defined as an independent socio-economic unit. Household members usually live in the same house or compound, pulling resources together to meet basic dietary and other vital needs with one person recognized as the head of the household. Individual members within the household can usually be related and identify themselves as belonging to the household. Trained field workers (enumerators) visit households in the HDSS area systematically to record births and deaths – the base of the vital events registration system – as well as in-and out-migration. Regular household visits were performed every three months until 2006 and every four months since then. Enumerators are supported by one key informant per village collecting vital events on a daily basis to ensure data accuracy locally. Each individual is assigned a unique identification number, which is maintained regardless of household rearrangement (e.g. marriage). Basic demographic parameters (date of birth, date of in- or out-migration, and date of death) are collected regularly. In addition, a questionnaire is administered that includes variables about religion, ethnic group, household characteristics, and individual members of the household, such as ID numbers of the parents (if a member of the HDSS). The parental ID allows identification of lineage and construction of relevant variables, such as age of parents at birth, birth order, or intervals between births.

### Overview on data handling

**Figure 1.**
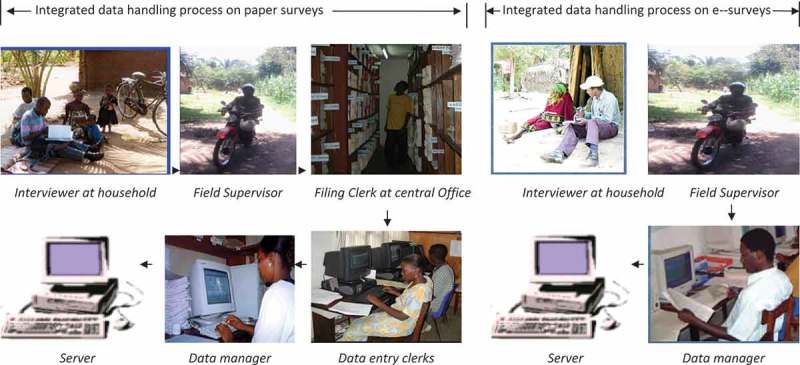
Integrated data collection and management cycle of household surveys in Rufiji Household and Demographic Surveillance *e-Surveys*, electronic surveys.

At each stage of the data-handling process, for both survey methods, the data are subject to quality checks until the data are thought to be sufficiently clean for archiving and analysis [13,22]. Figure 1 shows an integrated data collection and handling cycle of the RHDSS. The Rufiji HDSS team comprises administrative and support staff, one station manager, scientists, field supervisors, verbal autopsy supervisors, data managers, data entry clerks, filing clerk, and the enumerators. Details on the Rufiji HDSS procedures are described elsewhere [23,24]

### Study design

A total of 961 out of 16,000 active households were randomly selected to compare paper and electronic surveys. From December 2006 to February 2008, these households were visited separately by enumerators working on paper and electronic tools. In every quarter during the study period, the two surveys on the same household were organized such that paper surveys came first in half of the households and electronic surveys in the other half. For each household, the two enumeration sessions were at least 14 days apart to mitigate survey fatigue to household members. The enumerator who came last apologized for having another interview within a shorter period than the standard interval of 120 days for Rufiji HDSS.

As is the normal routine for HDSS, a log was kept of all staff involved and their actions at each stage of the data-handling process. Errors could be resolved by the enumerators, field supervisor, filing clerk at the site office, and members of the data-management team including entry staff, data manager, and their assistants. As far as possible, the version of the Household Registration System (HRS) was programmed on a mobile electronic device environment, BlackBerry Pearl 8130 (32-bit Intel XScale PXA272 312 MHz, microSD dedicated slot 64 MB), on top of a BlackBerry OS platform along with Java SE 6 and visual studio for back- and front-end platforms respectively. This helped to maintain tight quality controls throughout the processes. HRS is a software module used for handling HDSS data [24]. Enumerators were required to go through the entire set of forms for a particular household and individuals within it, and were responsible for entering data or issuing the skip command if there was no event in that particular household.

### Quality of data

A validation module on the HRS was run on a weekly basis to produce error reports from the datasets in either of the data-collection methods. The errors were categorized as (a) accuracy, logical, and consistency; (b) range; and (c) completeness and missing values. Examples of the first category include ‘birth event given to a male household member’, ‘household member is inactive, became migrated out but still has an event assigned to him’, ‘event date after date of interview’. The second category entailed scenarios of values outside the possibilities, for example: ‘an interview date not within date brackets of RHDSS visitation round’, ‘value other than those representing male and female is entered as someone’s sex’. The errors in the third category referred to the extent to which an expected attribute of data was provided such as ‘no value on one or more of the data variables’, ‘no record in an event file for a member of household for whom an event has been indicated on residence form’. Unique identifiers were issued to each error so that it was counted only once during analysis. Based on unique identifiers, re-emergence of the same error on subsequent reports due to failure of previous efforts to fix it could be spotted. The dates of error detection were recorded as well as when the form was sent back to the field for correction. We also recorded start and end dates of the error-correction process as well as when a household record was finally judged as ‘resolved’ or ‘unresolved’.

### Interview time

For paper surveys, two people recorded the beginning and end of an enumeration session using a stopwatch in 116 (10%) randomly selected households in the comparison study. In the case of a discrepancy greater than an arbitrary tolerance threshold of 5% of the duration of an interview, the time data were dropped and replaced with another household. The availability of time and funding jointly determined the size of the sample for stopwatch time recording. For electronic surveys, an internal clock of the device recorded the duration of enumeration sessions. In order to compare with stopwatch time data for paper surveys, 207 households surveyed on electronic tools were randomly selected and included in the analysis.

### Cost comparison

The cost data for paper and electronic surveys were obtained in two ways: (a) existing administrative records for the whole Rufiji HDSS during July 2007 to June 2008; and (b) logistics expenditures recorded during the course of the comparison study. Table 1 presents a summary of the parameters for which the costs per 1,000 households were proportionately deduced from the costs of the Rufiji HDSS as a whole as well as based on primary data that were directly recorded from households involved in the comparison study. Detailed costs in both cases are presented in Appendix 1.

**Table 1.** Cost items for household and demographic surveillance in Rufiji, Tanzania, during 2007/2008.

S. no.	Parameter	Paper surveys	Electronic surveys
1	Vehicles	SD	SD
2	Office equipment and furniture	SD	SD
3	Office and Storage	SD	SD
4	Computer equipment and software	SD	SD and PD
4.1	Purchase of PDA units	NA	PD
4.2	Customization of HRS to PDA environment	NA	PD
5	Networking	SD	SD
6	Communication	SD	SD
7	Field equipment	SD	SD and PD
8	Personnel costs – office team	SD	SD
9	Personnel costs – field team	SD	SD
10	Personnel costs – data-management team	SD	SD
11	Personnel costs – other	SD	SD
12	Training and supervision costs	SD	SD and PD
13	Transport – all other including maintenance except fuel	SD	SD
14	Transport – fuel and lubricants	SD	PD
15	Utilities (electricity, telephone, etc.)	SD	SD
16	Computing (antivirus subscription, software licence, etc.)	SD	SD and PD
17	Printing costs	SD	NA
18	Office (rent, photocopier maintenance, stationery, etc.)	SD	SD

SD (secondary data); came from whole of the Rufiji HDSS costs data and proportionally deduced for 1,000 comparison households; PD (primary data); were directly recorded from comparison study households; NA = not applicable; PDA = personal digital assistant; HRS = household registration system.

An example of the existing administrative records secondary data is the salary costs for enumerators and data managers, available from administrative records. An example of the primary data that were captured directly during the comparison study is the fuel costs incurred by field supervisors for their travel to resolve data errors and device faults.

Items with a life span of more than one year were considered to be capital goods, and their fixed costs were annualized to reflect real costs. The method for adjustment of expenditure to constant currency units [25] was used to convert costs from baseline to subsequent year. This was applied to work out the costs for 2008 for capital expenditure incurred before that as well as earlier than 2008 and also for conversion of the costs from 2008 to 2016. The indicative foreign-exchange market rate from the Central Bank of Tanzania at the midpoint of the comparison period was used to convert from Tanzanian shillings (TZS) to USA dollars (US$).

### Acceptance study

We interviewed 18 HDSS enumeration and observed enumeration sessions in 43 households. All but one of the qualitative interviews were carried out at the respondent’s household; the remaining one took place in a neighbouring residence. The qualitative interviewers observed and took notes from the moment when the enumerators arrived at the household to the end of the session. Fifteen household respondents who participated in the enumeration sessions were also interviewed. This was done after the DSS enumerator had left the household. The qualitative interviews focused on the effectiveness of the PDA compared with paper-based surveys, household, and enumerators’ perception of the changes and logistical challenges of managing the PDA. The qualitative interviewers had been trained to observe the interaction between enumerators, paying attention to verbal and symbolic reaction to the use of paper-based questionnaires and PDA. Enumerators and household members were then interviewed separately using guiding questions. Unstructured conversations between the enumerators and interviewers and between enumerators and household members were recorded and included in the qualitative study.

### Analysis

Visual FoxPro version 9 [26] and STATA version 12 [27] were used for data management and statistical analyses. There was one respondent per household, and most of the analyses were at household level, except in the qualitative inquiry for which an individual household respondent was the unit of analysis.

### Data-handling cycle

The number of staff involved throughout the HDSS processes and the number of error-correction actions at each stage were compared between electronic and paper surveys.

### Quality of data

The proportions of households with errors were compared between survey methods using McNemar’s test for paired data. Fisher’s exact test was used to assess the association between survey methods and the error types.

### Enumeration time

The Wilcoxon rank sum test was used to compare the interview duration between survey methods.

### Cost comparison

Costs from administrative records that were applicable to either of the survey methods were deduced to reflect an amount per 1,000 households. These were combined with logistic expenditure per 1,000 households, which were measured directly. To estimate the costs for 2015, the formula Expenditure in 2016 = Expenditure in 2008 × Deflator 2016/Deflator 2008 was applied with Deflator values of 209.5 for 2008 and 233.6 for 2016.

### Qualitative study

The data were transcribed and translated from Kiswahili to English, and coding was written manually. The main themes were identified from the data and operationalized throughout the analysis process beginning with interviews and supplemented with the field notes.

## Results

### Data-handling procedures

As illustrated in Figure 1, electronic surveys had three fewer people involved throughout the process and also three fewer error-checking points as the data moved back and forth between households and the server at main office.

### Quality of data

Table 2 presents the summary of errors detected in the datasets from paper and electronic surveys. Nine hundred and sixty-one active households during 2007–2008 were jointly visited by enumerators working on paper and electronic surveys. Using paper surveys, errors were detected in 166 (17%) households, and from electronic surveys, errors were detected in 15 (2%) households, p < 0.001. There were differences in the types of errors (p = 0.03). A higher proportion were due to accuracy in paper surveys (79%, 95% CI: 72%, 86%) compared with electronic surveys (58%, 95% CI: 29%, 87%). Errors in electronic surveys were more likely to be related to completeness (32%, 95% CI: 12%, 56%) than in paper surveys (11%, 95% CI: 7%, 17%).

**Table 2.** Proportion of households with errors on paper and electronic surveys, n = 961.

	Paper,n (%)	Electronic, n (%)	p value
Number of errors	172	19	–
Number of households with errors	166(17)	15(2)	<0.001^a^
Number of errors by types : Accuracy(logical, consistency)	136(79)	11(58)	
Values out of range	17(10)	2(11)	0.034*^b^*
Completeness or missing values	19(11)	6(32)	

^a^McNemar’s test for paired data.

^b^Fisher’s exact test for assessing association between survey methods and the types of errors.

### Duration of interviews

The median duration of an enumeration session per household was 9.4 minutes (90% central range 6.4, 12.2) for paper and 8.3 (6.1, 12.0) for electronic surveys (p = 0.001).

### Cost comparison

Table 3 presents a summary of the absolute and percentage differences in costs and cost-effectiveness between paper and electronic surveys per 1,000 households. Regardless of the quality of the data, electronic HDSS were less costly by 11% (US$ 1,010) than paper-based on total costs and 17% (US$ 1,070) on recurrent costs. For the error-free datasets, electronic HDSS were also less costly than paper-based by 28% (US$2,030) and 19% (US$ 2,230) on recurrent and total costs. For the error-free datasets, electronic HDSS were also less costly than paper-based by 28% (US$2,260) and 19% (US$ 2,490) on recurrent and total costs.

**Table 3.** Difference between paper and electronic surveys,in costs and cost-effectiveness of data per 1,000 households per year in RHDSS during 2007/2008.

	Paper	PDA	Difference (%)
**Error free dataset(cost–effectiveness)**			
Total costs	11.61	9.38	2.23(19)
Recurrent costs	7.33	5.31	2.03(28)
**Crude dataset**			
Personnel costs	3.82	3.14	0.68(18)
Recurrent costs (other than personnel)	2.45	2.20	0.24(10)
Recurrent costs (all)	6.27	5.20	1.07(17)
Capital costs	3.66	3.71	−0.05(1)
Total costs	9.93	8.91	1.01(10)

All values in US$ × 1,000 ; RHDSS = Rufiji Household and Demographic Surveillance System, Tanzania ; PDA = Personal Digital Assistant.

## Qualitative assessment

The use of the PDA did not seem to alter the enumeration process. DSS enumerators and household members exhibited a well-established familiarity to each other. Upon arrival at the household, enumerators and residents would exchange greetings often with jokes, and soon the enumerators would proceed to enquire about demographic information. Where the PDA was used for the first time, enumerators would typically inform the household that the new electronic device was a replacement of the questionnaire in collecting the routine information and gave a briefing on how it works. No household respondents indicated resistance to the use of the PDA, and in many cases enumerators were not subjected to extensive interrogation. In some cases, the enumerators would let the respondents have a look at the PDA, when requested to do so, but it would not take long before it was handed back and the survey started. In most cases, the respondents would ask about how the PDA works; the same questions they used to hear were entered into the device, with data transfer and what to do when it does not work properly. They also asked other questions not related to the device such as the rationale for routine collection of the surveillance data. Interviews with the enumerators revealed that the questions on the use of the demographic data were about the device itself since it was common even before they started using the PDAs for HDSS. The following was extracted from the observation notes in a household where a PDA was used.

‘At the beginning of the process, the respondent stood very close to the enumerator paying attention to the PDA as she listened and responded to the questions but after some few questions she started doing other activities. She also went in the house more than three times during the interview session.’

From interviews with enumerators and observation, the practice of ‘doing routine chores’ while responding to the enumerators was a familiar practice. The notes further reveal that in many cases, respondents would continue doing activities such as washing dishes, cooking, and breastfeeding as they responded to the enumerator’s questions (source notes from informal conversations).

Questions were considered by most of the HDSS respondents to be more or less the same as in previous visits, with the exception of the use of electronic devices with which they seemed to be unfamiliar. This was regardless of the survey method. Three respondents, among those for whom HDSS surveys on electronic devices were observed, asked specific questions about the electronic device. The questions were related to how it functioned, what the researchers did with the recorded data, and whether the device was recording voice as well.

## Enumerators’ perceptions about the effectiveness of the PDA

Enumerators were overwhelmingly in favour of using a PDA, as it was found to be practically convenient, being easy to carry and protect against the elements, for instance, when it was raining. The automated skip function was seen as advantageous and time-saving, since the enumerators did not have to read through those questions in every visit to the same household. Enumerators pointed that the devices were exciting, interesting, and prestigious, and were seen as skilled professionals in the eyes of the community. Both enumerators and household respondents perceived the sessions to be shorter and questions straightforward. However, enumerators saw deficiencies in the PDA. For instance there gave examples of rare cases where the device would stop functioning and enumerators lost their productive time until the problem was solved. Depending on severity of the problem, ‘trouble shooting’ was done either by the enumerator or by the data manager. Enumerators’ waiting time for device recovery could be anything from half an hour to several hours, or the next day, depending on the distance from the specific household to the office and nature of the problem. For such scenarios, enumerators that thought papers would sometimes guarantee a productive working day compared with PDAs.

## Perceptions on completeness and quality of records

PDA was associated with data completeness. In paper-based enumeration sessions, enumerators were observed skipping questions, especially the demographic information and instead go directly to details. Sometimes they just asked ‘So tell me what is new here’. In such instances, household respondents mentioned the relevant HDSS events spontaneously. This was common in paper surveys and was supported by household respondents and enumerators as follows:

‘Most of the questions have been the same for years and sometimes they start telling you about all the events that happened at their homestead even before you asked them, they know what we are going to ask for.’ (interview with enumerator)

‘We know all these questions; we know the enumerators as well. They come to us every now and then so nothing new today, may be the PDA.’ (interview with household respondent)

In some cases, enumerators would record only some of the information and moved to the next home, hoping to fill the rest of details later after the working day.

‘Sometimes you have to take a few notes at the field but you need to complete the records later at home, especially for households with many events to update.’ (interview with enumerator)

Other issues, raised by enumerators and household respondents about their opinion and perception regarding the survey methods under review are summarized in Appendix 2.

## Discussion

We have presented the comparative experiences of HDSS surveys using paper and electronic tools. As in previous studies, our findings support the use of electronic tools to address some of the known challenges of data management in traditional paper-based health surveys. They include a reduction in turnaround time for results availability, improvements on quality of data, and cost savings [28,29]. We found that electronic surveys, compared with the paper method, involved fewer staff, shortened survey procedures, and improved quality of data at lower costs. Moreover, there was a good acceptance of both methods by the enumerators and household respondents, even though electronic tools were characterized by technical problems during the enumeration sessions. Our study also shows that PDAs’ advantages go beyond the technical merits, regarding the motivation of the field workers enhancing the sense of ownership and accountability to their job. PDA functions such as ‘skip’ may help enumerators work faster but will not grant the ‘freedom’ to say ‘tell me what is new here’ when they visit households. For this case, the longevity of the interest in PDA is yet to be established. Programme managers might need to renovate mechanisms for motivating the field workers to avoid boredom. Some information required for surveillance purposes has emotional and ethical implications. HDSS has so far been a unilineal information flow from the community to the programme [30]. There is already an indication that ethical issues may need to be better managed in RHDSS, e.g. through capacity building of enumerators, and better communication between the programme and community. Silence on the part of the programme may jeopardize the process in the future, regardless of the effectiveness of data-collection tools despite the known potential of mHealth interventions for public health surveillance, and recent reviews have highlighted a scarcity of evidence in the sub-Saharan Africa region context [31,32]. The main contribution of our findings therefore is the evidence for the feasibility of electronic tools for continuous longitudinal surveillance systems.

We foresee that electronic tools will be useful for HDSSS as well as for the Sentinel Panel of Districts (SPD), which provide nationally representative data on demographic and health indicators and Sample Vital Registration with Verbal Autopsy, part of the SPD for the generation of nationally representative estimates of mortalities based on age, sex, residence, and zone [33]. There is a considerable lack of effective and comprehensive civil registration and vital statistics systems particularly in developing countries [12] such that there is a need to evaluate alternative approaches towards improving the situation. Currently there are at least 52 HDSS sites in 20 countries across Africa, Asia, and Oceania [34]

In addition to the required supply of information from a variety of sources including the HDSS platforms, there are issues of system costs as well as coverage and representativeness of the survey platform. According to Setel et al. [3], costs should be considered when policy-makers and programme planners are making investment choices for HIS, but costs are rarely known. We observed, with electronic surveys, an elimination of three data-processing steps as well as three fewer people involved in the processes, compared with paper tools. There were also significant savings of time during interviews at each household, which are likely to be of public health importance when the cumulative magnitude is considered. There was also, with electronic surveys, a 28% reduction in recurrent costs compared with paper-based HDSS. Such gains provide an opportunity for optimizing the utilization of resources.

## Conclusion

To be of public health utility and especially in limited resource settings, electronic data collection must demonstrate potential on the main pillars of data quality. From the experience in Rufiji HDSS, the PDAs have shown a reduction in costs as well as an improvement in the completeness, accuracy, and timelines of the survey data. The research team hopes that this approach will become a new standard in the HSs in Tanzania and elsewhere in the developing countries.

## Paper context

Most contemporary survey and longitudinal surveillance studies have migrated from paper-based methods to electronic data collection on mobile devices. This choice has been largely justified for practical reasons. However there have been few direct comparative studies of the relative quality, time and cost of the results generated by the two approaches. This paper contributes empirical findings indicating superior performance of electronic data collection across all criteria in an African setting.

## Appendix

**Appendix 1.** Annualized capital costs for Household Demographic surveillance systems surveys per Household in Rufiji Tanzania, during 2007–2008 for paper and electronic devices.

	Paper tools	PDA-based tools
Cost items	Annualized cost /1,000 HHs	Annualized cost /1,000 HHs
Vehicles, motorcycles, bicycles	686.6	686.6
Photocopy machine and furniture cutter/binder, big filing cabinet safe,chairs, shelves, desks	56.6	45.3
Container for storage and office renovations	171.9	171.9
Computers and accessories, programming, printers	713.3	797
Software, networking, communication equipment	3,113.7	3,073.3
Field equipment’s	61.9	61.9
Personnel costs office team	1,369.7	1,369.7
Personnel costs field team	1,021.1	1,021.1
Personnel costs data room team	1,142	462.5
Other personnel driver/mechanic, cleaner, watchman	289	289
Training and supervision (update round retraining, periodic meetings)	296.4	296.4
Transport	1,437.9	1,437.9
Utilities, electricity, fuel, stationary, computer-related	3,099.4	2,604.1

HHs, households; PDAs, personal digital assistants; HDSS, household demographic surveillance systems.

**Appendix 2.** Summary of the advantages and disadvantages of electronic surveys as perceived or observed by interviewers and respondents in RDSS.

Interviewer’s perception (author’s opinion in brackets)		Respondent’s perception (author’s opinion in brackets)	
Advantage	Disadvantage	Advantage	Disadvantage
Time saving as sessions were shorter, and questions were straightforward (this is likely to mitigate interview fatigue in respondents as well as interviewers)	Device would stop functioning before session came to an end (unexciting, tiresome, and could ruin credibility of the DSS process); in the long run, this could lessen the eagerness of the community to participate	Time saving, as sessions were shorter, and questions were straightforward	Device would stop functioning before the session came to an end
New experience in which we had to record all survey data on a survey tool right on the spot. On traditional paper-based surveys, we were used to recording some of the responses on a separate sheet and transferring them onto a survey tool later when we returned home. This was mainly true in the case of a large number of members and events in a particular household. (With the new practice, it is likely to mitigate the original twofold window for data-transcription errors, which is the case on traditional paper-based surveys.) Transcription errors were likely to happen at the time of the interview and then while transferring of data from original sheet to an actual survey tool.			
Automatic retrieval of respondent’s particulars and for all other members of the HH. On traditional paper-based surveys, the interviewers had to re-write the particulars of household members on fresh event forms. (In the long run for the whole survey as well as at the level of one household, this might lead to time-savings for other tasks.)		Automatic retrieval of respondent’s particulars and for all other members of the HH. On traditional paper-based surveys, the interviewers had to re-write the particulars of household members on fresh event forms.	
More exciting, interesting, prestigious and convenient to carry and use to carry. (Excitement and prestige are likely to improve motivation of interviewers.) However this could only be at first experience and therefore short-lived. Convenience for carrying and use would reduce logistics fatigue and create more time for interviewers on issues related to quality of data rather than logistics.		Interesting to see our information entered into a computer right in front of us. (Excitement by respondents is likely to reduce interview fatigue.)	
Presence and efficiency of skip function would ultimately improve sense of data quality among interviewers.

**Appendix 3.** Summary of the advantages and disadvantages of paper-based surveys as perceived or observed by interviewers and respondents in RDSS.

Interviewer’s perception (author’s opinion)		Respondent’s perception	
Advantage	Disadvantage	Advantage	Disadvantage
Unspoken but common understanding between interviewers and respondents that ‘you know the questions’ or ‘we know the answers you need’ (It makes the survey more prone to skipping of questions and in turn jeopardize overall data integrity.)	Recording people’s particular afresh on paper leads to more time needed to manual resolving of consistency of data across households and members within the same household		
	Many papers to carry and much hardre to protect them in case of rain (contributes to survey fatigue to interviewers)		
	Double data entry creates need for many more contacts with data room for resolving data consistency issues (increases the chance of data errors on new event forms particularly on people’s permanent IDs)	It leads to more time needed to resolve an error related to consistency of data	
It allows partial recording of data while at a household. Other information goes on lose sheet for later transfer on dedicated survey form. (This makes survey values more prone to recall effect, as interviewers might not be able to recall them all and precisely).			
